# Coenzyme Q_10_ Reduces Infarct Size in Animal Models of Myocardial Ischemia-Reperfusion Injury: A Meta-Analysis and Summary of Underlying Mechanisms

**DOI:** 10.3389/fcvm.2022.857364

**Published:** 2022-04-15

**Authors:** Kamal Awad, Ahmed Sayed, Maciej Banach

**Affiliations:** ^1^Faculty of Medicine, Zagazig University, Zagazig, Egypt; ^2^Zagazig University Hospitals, Zagazig, Egypt; ^3^Faculty of Medicine, Ain Shams University, Cairo, Egypt; ^4^Department of Preventive Cardiology and Lipidology, Chair of Nephrology and Hypertension, Medical University of Lodz (MUL), Lodz, Poland; ^5^Department of Cardiology and Adult Congenital Heart Diseases, Polish Mother's Memorial Hospital Research Institute (PMMHRI), Lodz, Poland; ^6^Cardiovascular Research Centre, University of Zielona Gora, Zielona Gora, Poland

**Keywords:** ubiquinone, animals, rats, coenzyme Q10, pre-clinical, myocardial reperfusion injury

## Abstract

**Objective:**

Effective interventions that might limit myocardial ischemia-reperfusion (I/R) injury are still lacking. Coenzyme Q_10_ (CoQ_10_) may exert cardioprotective actions that reduce myocardial I/R injury. We conducted this meta-analysis to assess the potential cardioprotective effect of CoQ_10_ in animal models of myocardial I/R injury.

**Methods:**

We searched PubMed and Embase databases from inception to February 2022 to identify animal studies that compared the effect of CoQ_10_ with vehicle treatment or no treatment on myocardial infarct size in models of myocardial I/R injury. Means and standard deviations of the infarct size measurements were pooled as the weighted mean difference with 95% confidence interval (CI) using the random-effects model. Subgroup analyses were also conducted according to animals' species, models' type, and reperfusion time.

**Results:**

Six animal studies (4 *in vivo* and 2 *ex vivo*) with 116 animals were included. Pooled analysis suggested that CoQ_10_ significantly reduced myocardial infarct size by −11.36% (95% CI: −16.82, −5.90, *p* < 0.0001, I^2^ = 94%) compared with the control group. The significance of the pooled effect estimate was maintained in rats, Hartley guinea pigs, and Yorkshire pigs. However, it became insignificant in the subgroup of rabbits −5.29% (95% CI: −27.83, 17.26; I^2^ = 87%). Furthermore, CoQ_10_ significantly reduced the myocardial infarct size regardless of model type (either *in vivo* or *ex vivo*) and reperfusion time (either ≤ 4 h or >4 h).

**Conclusion:**

Coenzyme Q_10_ significantly decreased myocardial infarct size by 11.36% compared with the control group in animal models of myocardial I/R injury. This beneficial action was retained regardless of model type and reperfusion time.

## Introduction

Ischemic heart disease (IHD) is still the leading cause of death worldwide (1). A total of 197 million cases and 9.14 million deaths of IHD have been recorded globally in 2019 (1). Acute myocardial infarction (AMI) is the most serious form of IHD that is caused by decreased or complete cessation of the blood supply to a part of the cardiac muscle leading to ischemia and infarction of the affected portion. The American Heart Association (AHA) estimates that one American will experience an event of AMI nearly every 40 s and about 14% of AMI cases will result in death (2). Despite the progress in myocardial reperfusion methods over the past decade (e.g., pharmacological thrombolysis and percutaneous coronary intervention [PCI]), the mortality and morbidity associated with AMI and its sequelae (e.g., heart failure [HF]) are still significant (3, 4). One important flaw of reperfusion strategies is the development of myocardial ischemia-reperfusion (I/R) injury, which eventually constitutes up to 50% of the infarct size (3, 5). The exact pathophysiology of I/R injury is not completely understood (6). However, possible underlying mechanisms include mitochondrial damage, oxidative stress, inflammation, and excess calcium (7–9). Infarct size is a crucial prognostic factor in patients with AMI (10). Therefore, there is a large need for effective cardioprotective approaches that aim for infarct size limitation.

Coenzyme Q_10_ (CoQ_10_), also known as ubiquinone, is a lipophilic benzoquinone that presents in the cell membranes all over the body, particularly in the mitochondria (11). It plays a key role in ATP production through the electron transport chain and oxidative phosphorylation (12). It also exhibits antioxidant and membrane-stabilizing functions inside and outside the mitochondria (13, 14). Since the heart is a very active organ that requires much energy, it normally contains high levels of CoQ_10_ (15). Low levels of myocardial CoQ_10_ have been observed in many cardiac diseases such as IHD, cardiomyopathy, and chronic HF (16–19). In multiple reports, CoQ_10_ reduced creatine kinase (CK) leakage during myocardial I/R injury (20–22). Therefore, CoQ_10_ may represent a promising cardioprotective agent in case of cardiac I/R injury. We conducted this systematic review and meta-analysis to assess the potential cardioprotective effect of CoQ_10_ and its related molecular mechanisms in myocardial I/R injury in animal studies.

## Methods

We followed the preferred reporting items for systematic reviews and meta-analyses (PRISMA) during this study preparation (Supplementary Table 1). The protocol of this meta-analysis was not prospectively registered.

### Literature Search Strategy

We systematically searched both PubMed and Embase databases from inception to February 2022 using a combination of related keywords and MeSH terms as follows: (Coenzyme Q_10_ OR CoQ_10_ OR Ubiquinone OR “Ubiquinone”[Mesh]) AND (infarct OR infarction OR myocardial infarction OR myocardial injury OR myocardial necrosis OR myocardial death OR “Myocardial Infarction”[Mesh]) AND (size OR area OR region OR part OR portion OR zone). We did not use any restriction filters throughout the search. We also manually searched related review articles for potential missing studies (23).

### Inclusion and Exclusion Criteria

Experimental studies were included if they met the following predefined criteria: (1) being an animal study on experimental models of myocardial I/R injury (either *in vivo* or *ex vivo*), (2) compared CoQ_10_ (either ubiquinol or ubiquinone form) with vehicle treatment or no treatment, and (3) data on myocardial infarct size, defined as the percentage of infarct zone over the area at risk or the total ventricular myocardium, were reported in both groups.

Exclusion criteria were as follows: (1) *in vitro* studies, (2) studies that included animals with cardiovascular (CV) comorbidities (e.g., obesity and diabetes mellitus), (3) studies with non-English or inaccessible text, (4) retracted studies that contained false or fabricated data, and (5) studies that missed any of the inclusion criteria.

### Data Extraction

The following information was extracted from each study: (1) first author's name, (2) publication year, (3) study location, (4) animal characteristics (i.e., species, weight, age, and sex), (5) treatment group characteristics (i.e., sample size, vehicle type, intervention dose, duration, and route of administration), (6) type of used anesthesia, (7) methods of model preparation, (8) data on the infarct size in each group, and (9) data on secondary outcomes assessing the cardiac function that included left ventricular ejection fraction (LVEF), LV developed pressure (LVDP), and LV dP/dtmax.

### Risk of Bias Assessment

The risk of bias was assessed using the SYRCLE's risk of bias tool (24). This tool includes 10 domains as follows: (1) sequence generation, (2) baseline characteristics, (3) allocation concealment, (4) random housing, (5) blinding of the investigator, (6) random outcome assessment, (7) blinding of the outcome assessor, (8) incomplete outcome data, (9) selective outcome reporting, and (10) other sources of bias. Each domain is judged either the low, high, or unclear risk of bias. Disagreements were settled by discussion.

### Quantitative Data Synthesis

Means and standard deviations (SDs) of the infarct size measurements in the included studies were pooled as weighted mean difference (WMD) with 95% confidence interval (CI) using the DerSimonian and Laird random-effects model to address inter-study heterogeneity. If one of the included studies reported standard error (SE) instead of SD, we calculated SD using the formula: SD = SE × square root (sample size) (25). Heterogeneity was judged by visual inspection of the generated forest plot and measured by both I^2^ and χ^2^ tests. To test the result's robustness, leave-one-out sensitivity analysis was applied by removing one study successively and performing the analysis again. To address potential heterogeneity, we also conducted subgroup analyses according to animals' species, models' type (either *in vivo* or *ex vivo*), and reperfusion time (either ≤ 4 h or >4 h).

Publication bias was judged by Begg's funnel plot and Egger's test (26). In addition, the *trim and fill* approach was applied in the case of an asymmetrical funnel plot to adjust for the potential publication bias (27). All analyses were done by RevMan version 5.3 (The Cochrane Collaboration, Oxford, UK) and Comprehensive Meta-Analysis version 3 (Biostat, New Jersey, USA).

## Results

### Flow and Characteristics of Included Studies

**Figure 1:**
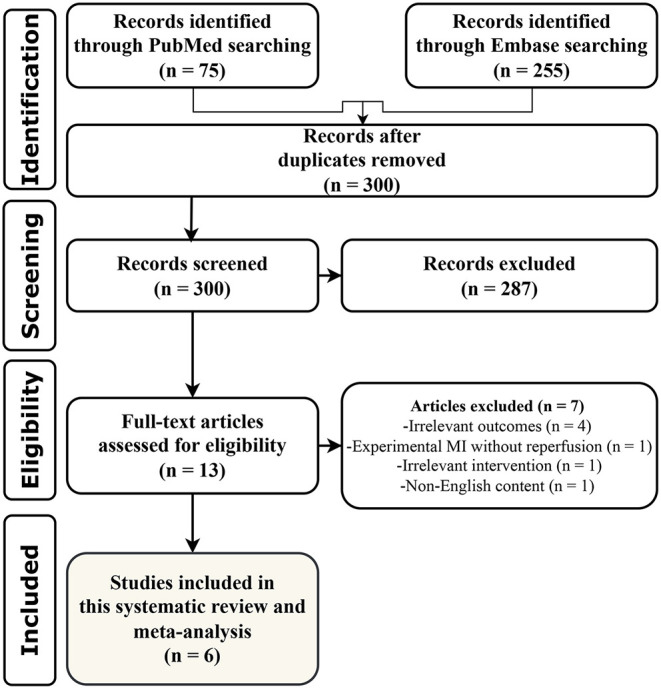
PRISMA flow diagram of study screening and selection process. MI, myocardial infarction.

We identified 330 records through the literature search. Of them, 300 records remained after the exclusion of duplicates. Initial title/abstract screening resulted in 13 potentially relevant studies. Following the more detailed screening of their full texts, 6 relevant animal studies (4 *in vivo* and 2 *ex vivo*) were finally included in this systematic review and meta-analysis (28–33). All steps of study selection are summarized in Figure 1.

A total of 116 animals were enrolled in the included studies. Each relevant group (i.e., CoQ_10_ and control groups) included 58 animals. Four included studies (28, 30, 32, 33) were conducted in the United States, one (31) in China, and one (29) in India. The year of publication ranged from 1996 to 2017. Two of the included studies (28, 33) used New Zealand White rabbits, two used (29, 31) rats (Sprague Dawley or Wistar), one (30) used Hartley guinea pigs, and one (32) used Yorkshire pigs. All included animals were males. Ischemia was induced by blockage of the left anterior descending (LAD) artery in two studies (31, 32), the left main coronary artery in two studies (29, 33), the left circumflex artery in one study (28), and the aorta/atria in one study (30). Infarct size was reported in all included studies as a percentage of the area at risk except for one study as a percentage of the total left ventricle (32). More details on the characteristics of the included studies are shown in Table 1.

**Table 1:** Characteristics of the included studies.

**References**	**Country**	**Species (sex)**	**Weight**	**Model type**	**Anesthetic**	**Method of ischemia**	**Duration of I/R**	**Groups of interest**	**Time**	**Approach**
Birnbaum et al. (28)	USA	NZW rabbits (M)	2 to 3.6 kg	*in-vivo*	Ketamine/xylazine	Blockage of LCX or anterolateral branch of it	30 min/4 h	Coenzyme Q10 30 mg (*n* = 10)	After 13 min of ischemia	IV infusion
								Placebo 12 ml (*n* = 10)		
								Coenzyme Q10 30 mg (*n* = 6)	60 min before ischemia	
								Placebo 12 ml (*n* = 6)		
Khan et al. (29)	India	Wistar rats (M)	200 to 250 gm	*in-vivo*	NR	LCA blockage	30 min/ 45 min	Coenzyme Q10 1 mg/kg (*n* = 6) Control (*n* = 6)	Before I/R injury induction (for 7 days)	NR
Lekli et al. (30)	USA	Hartley guinea pigs (M)	350 to 400 gm	*ex-vivo*	Sodium pentobarbital	Clamping of atrial and aortic cannulas	30 min/ 120 min	Coenzyme Q10 5 mg/kg (*n* = 6) Vehicle (*n* = 6)	Before I/R injury induction (for 30 days)	Gavaging
Liang et al. (31)	China	SD rats (M)	250 (10) gm	*in-vivo*	Sodium pentobarbital	LAD ligation	45 min/ 72, 24 and 2h	Coenzyme Q10 6 mg/kg/mL (*n* = 6)^*^	3 days Before I/R induction	IP
								Soybean oil solvent (*n* = 6)^*^		
Maulik et al. (32)	USA	Yorkshire pigs (M)	18 to 25 kg	*ex-vivo*	Sodium pentobarbital	LAD ligation	15 min/ 120 min	Coenzyme Q10 5 mg/kg (*n* = 6) Placebo (*n* = 6)	Before I/R injury induction (for 30 days)	NR
Verma et al. (33)	USA	NZW rabbits	2.5 to 3.5 kg	*in-vivo*	Ketamine/xylazine	LCA blockage	30 min/3 h	Coenzyme Q10 liposomes 36 mg (*n* ≈ 6)	Before I/R induction	Intracoronary infusion
								Empty liposomes (*n* ≈ 6)		

*I/R, ischemia reperfusion; NZW, New Zealand White; SD, Sprague Dawley; IV, intravenous; IP, intraperitoneal; NR, not reported; M, males; LCX, left circumflex artery; LCA, left coronary artery; LAD, left anterior descending artery*.

* *In each group according to the different durations of reperfusion*.

### Risk of Bias in the Included Studies

According to SYRCLE's risk of bias tool, all included studies showed a low risk of bias concerning the following three domains: baseline characteristics, selective outcome reporting, and other sources of bias. No studies reported any information about sequence generation, allocation concealment, random housing, blinding of the investigator, or random outcome assessment. One study by Liang et al. (31) reported blinding of the outcome assessor. A study by Khan et al. (29) was at a high risk of bias due to incomplete outcome data reporting. The risk of bias assessment is summarized in Table 2.

**Table 2:** Summary of the risk of bias assessment of the included studies.

**Study/domain**	**Sequence generation**	**Baseline characteristics**	**Allocation concealment**	**Random housing**	**Blinding of the Investigator**	**Random outcome assessment**	**Blinding of the outcome assessor**	**Incomplete outcome data**	**Selective outcome reporting**	**Other sources of bias**
Birnbaum et al. (28)	Unclear	Low	Unclear	Unclear	Unclear	Unclear	Unclear	Low	Low	Low
Khan et al. (29)	Unclear	Low	Unclear	Unclear	Unclear	Unclear	Unclear	High	Low	Low
Lekli et al. (30)	Unclear	Low	Unclear	Unclear	Unclear	Unclear	Unclear	Low	Low	Low
Liang et al. (31)	Unclear	Low	Unclear	Unclear	Unclear	Unclear	Low	Low	Low	Low
Maulik et al. (32)	Unclear	Low	Unclear	Unclear	Unclear	Unclear	Unclear	Low	Low	Low
Verma et al. (33)	Unclear	Low	Unclear	Unclear	Unclear	Unclear	Unclear	Low	Low	Low

### Meta-Analysis Results Regarding Myocardial Infarct Size

**Figure 2:**
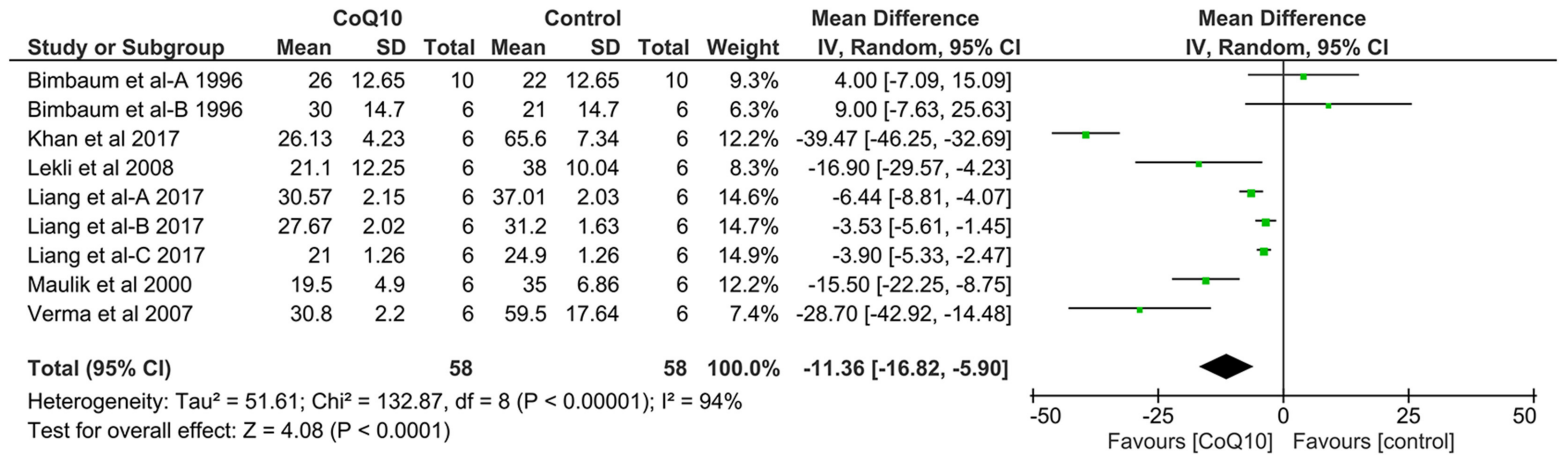
Forest plot displaying the results of meta-analysis of coenzyme Q_10_ effect on myocardial infarct size in models of myocardial ischemia-reperfusion injury compared with the control group. CI, confidence interval; df, degrees of freedom; SD, standard deviation; IV, inverse variance.

Pooled analysis of 6 studies including 116 animals revealed that CoQ_10_ significantly reduced myocardial infarct size by −11.36% (95% CI: −16.82, −5.90, *p* < 0.0001, Figure 2) compared with the control group in experimental models of myocardial I/R injury. Significant between-study heterogeneity was observed in this meta-analysis (χ^2^ = 132.87, *p* < 0.00001, I^2^ = 94%). The significance of the pooled effect estimate did not alter when we applied the leave-one-out sensitivity analysis, indicating the robustness of the observed result.

### Subgroup Analysis Results

In subgroup analysis, according to included animal species, the significance of the pooled effect estimate was maintained in rats, Hartley guinea pigs, and Yorkshire pigs. However, it became insignificant in the subgroup including rabbits −5.29% (95% CI: −27.83, 17.26; I^2^ = 87%; Table 3). Moreover, CoQ_10_ significantly reduced the myocardial infarct size regardless of model type (either *in vivo* −10.14% [95% CI: −16.22 to −4.07] or *ex vivo* −15.81% [95% CI: −21.76 to −9.85]) and reperfusion time (either ≤ 4 h −13.43% [95% CI: −26 to −0.85] or >4 h −4.93% [95% CI: −7.78 to −2.08]).

**Table 3:** Summary of subgroup analyses results.

**Subgroups**	**No. of comparisons**	**MD (95% CI)**	**I^**2**^**	**Chi^**2**^, *P* value**	***P* value for interaction**
**Species**					0.737
Rats	4	−12.05 (−18.98 to −5.12)	97%	<0.0001	
Rabbits	3	–**5.29 (**–**27.83 to 17.26)**	87%	<0.0001	
Hartley guinea pigs	1	−16.9 (−29.57 to −4.23)	NA	NA	
Yorkshire pigs	1	−15.5 (−22.25 to −8.75)	NA	NA	
**Models type**					0.192
*In vivo*	7	−10.14 (−16.22 to −4.07)	95%	<0.0001	
*Ex vivo*	2	−15.81 (−21.76 to −9.85)	0	0.848	
**Reperfusion time**					0.196
≤ 4 h	7	−13.43 (−26 to −0.85)	95%	<0.0001	
>4 h	2	−4.93 (−7.78 to −2.08)	70%	0.07	

*MD, mean difference; NA, not applicable. Bold values indicates that the result became insignificant*.

Significant heterogeneity was observed in all studied subgroups except for the subgroup included *ex vivo* studies (I^2^ = 0%; χ^2^, *p* = 0.848). All details on subgroup analyses are summarized in Table 3.

### Results Regarding Cardiac Function Parameters

Data on LVEF were reported only in the study by Liang et al. (31). Significant improvement in LVEF was observed in CoQ_10_ group (mean: [SD] 67.12 [6.18]) compared with the control group 59.12 (5.81). Data on LVDP were reported in the studies by Lekli et al. (30) and Maulik et al. (32), while data on LV dP/dtmax were reported in the studies by Maulik et al. (32) and Liang et al. (31). Recovery of LVDP and LV dP/dtmax was significantly better in CoQ_10_ group compared with the control group. The summary of data on cardiac function outcomes is shown in Table 4.

**Table 4:** Summary of results on cardiac function parameters.

**Studies**	**Lekli et al**. **(****30**)^*^		**Maulik et al**. **(****32**)^*^		**Liang et al**. **(****31**)^**^	
**Outcomes/Groups**	**CoQ10 group^†^**	**Control group**	**CoQ10 group^†^**	**Control group**	**CoQ10 group^†^**	**Control group**
LVEF (%)	NR		NR		67.12 (6.18)	59.12 (5.81)
LVDP (mmHg)	64 (3)	45 (3)	131 (4.2)	92 (3.9)	NR	
LV dP/dtmax (mmHg/ms)	NR		1.976 (0.085)	1.11 (0.098)	2.25 (0.12)	1.84 (0.08)

*LVEF, left ventricular ejection fraction; LVDP, left ventricular developed pressure; NR, not reported*.

*All data are presented as mean (standard error) except for data by Liang et al. are presented as mean (standard deviation)*.

* *After 120 min of reperfusion*.

** *After 72 h of reperfusion*.

† *p <0.05 compared with the control group*.

### Publication Bias

**Figure 3:**
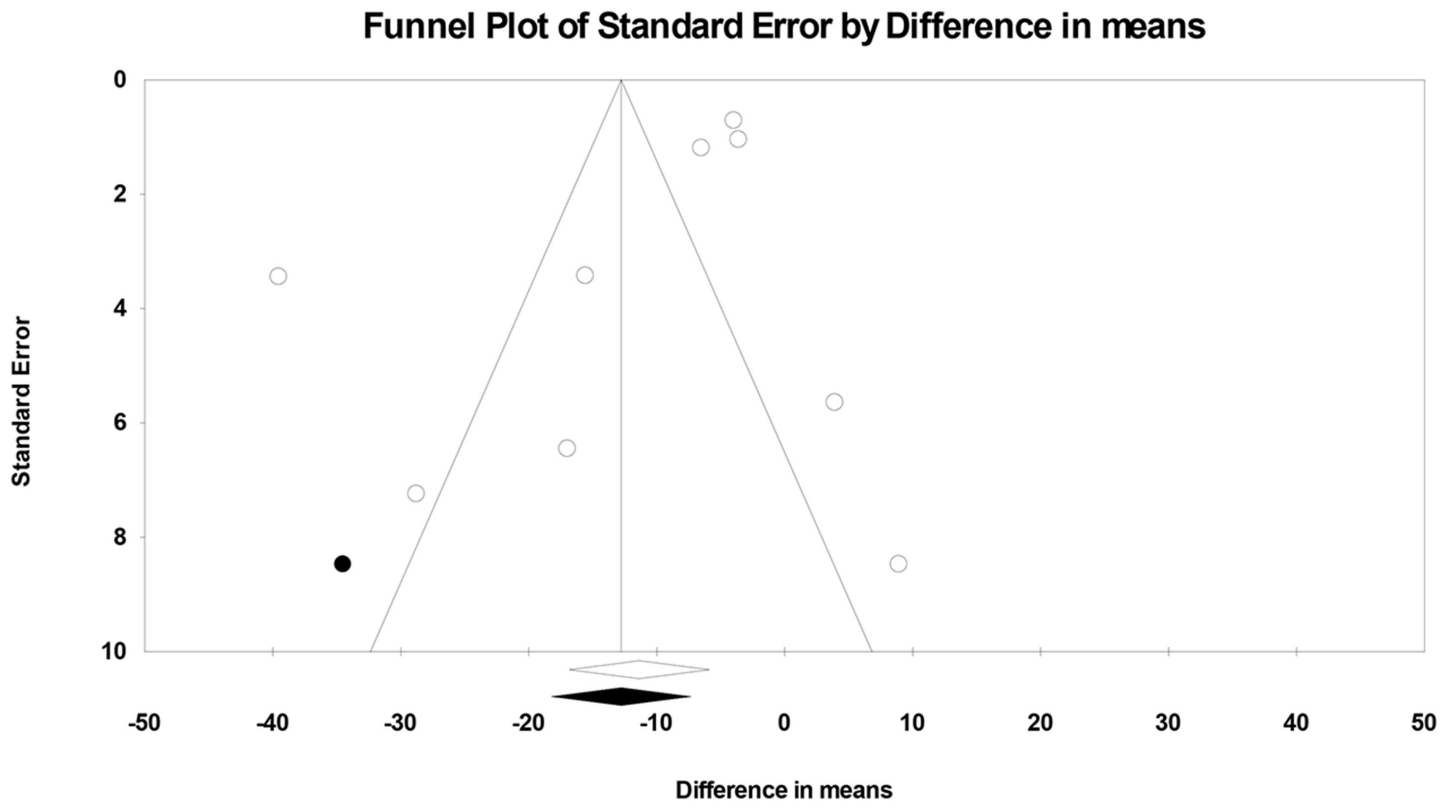
Corrected funnel plot showing publication bias in animal studies that compared the effect of coenzyme Q_10_ with vehicle treatment or no treatment on myocardial infarct size in models of myocardial ischemia-reperfusion injury.

Visual inspection of the generated funnel plot suggested potential publication bias in the observed result (Figure 3). The *trim and fill* approach adjusted the pooled effect estimate for this potential bias as follows: −12.78% (95% CI: −18.23, −7.32) by imputing one study to the left of its mean. The significance of the effect estimate was not changed after this adjustment. On the contrary, Egger's test detected insignificant publication bias in the current analysis (two-tailed *p*-value = 0.18).

## Discussion

### Evidence Summary

To the best of our knowledge, this is the first meta-analysis to explore the potential cardioprotective limiting effect on infarct size of CoQ_10_ in myocardial I/R injury. Our meta-analysis of 6 studies including 116 experimental models of myocardial I/R injury suggested that CoQ_10_ significantly decreased myocardial infarct size by 11.36% compared with the control group. In addition, this beneficial effect was preserved irrespective of model type (either *in vivo* or *ex vivo*) and reperfusion time (either ≤ 4 h or >4 h). Likewise, the significant improvement of cardiac function parameters (e.g., LVEF and LVDP) with CoQ_10_ was observed in multiple included studies. These results are of interest in the context of involved molecular mechanisms and their implications in informing future research on the promise of CoQ_10_ as a cardioprotective agent.

### Underlying Molecular Mechanisms

There are a number of different mechanisms, which may account for the cardioprotective effect of CoQ_10_ seen in our analysis. First, CoQ_10_ has been shown in numerous studies to act as an antioxidant, increasing the levels of superoxide dismutase and glutathione and decreasing the levels of lipid peroxidation (31). This antioxidative activity is crucial as oxidative stress is believed to play a significant role in myocardial I/R injury (3). Following reperfusion of an ischemic heart, there is an increased production of free oxygen radicals that induces further cellular damage. Specifically, dysfunction of the mitochondrial electron transport chain results in the increased production of free oxygen radicals (34). These radicals then damage cardiolipin, an important component of the inner mitochondrial membrane (35). Cardiolipin damage may precipitate further leakage of electrons from the mitochondria (36), leading to the formation of greater amounts of superoxide anion radicals, therefore precipitating a vicious cycle that causes severe cellular damage (37). In multiple studies, CoQ_10_ inhibited oxidative inactivation of CK and reduced its leakage during myocardial I/R insult (21, 22, 38, 39).

Additionally, CoQ_10_ has been shown to reduce the levels of p53 (31), which is a well-recognized pro-apoptotic protein (40). P53 exerts its pro-apoptotic effect by enhancing the transcription of a group of pro-apoptotic members of the bcl-2 family, named BH3-only proteins (40–42). These proteins inhibit the anti-apoptotic members of the bcl-2 family and may enhance other pro-apoptotic bcl-2 proteins such as BAX and BAK (43). Therefore, by inhibiting the activity of p53, CoQ_10_ may reduce cellular apoptosis and thus reduce infarct size. In addition to its ability to diminish pro-apoptotic activity, CoQ_10_ has also been shown to increase the gene expression of anti-apoptotic bcl-2, leading to decreased apoptotic activity and preserved cellular structures in the setting of I/R injury (29). Besides, Khan et al. (29) observed that CoQ_10_ reduced apoptotic DNA levels through inhibition of caspase-9 and cytochrome-C release into the cytoplasm.

It has been reported that CoQ_10_ increased the levels of adenosine triphosphate (ATP) and creatine phosphate and enhanced the aerobic efficiency of the myocardium in I/R injury (31, 39). Increased production of nitric oxide was also observed with CoQ_10_ resulting in coronary vasodilatation (29).

Autophagy is a vital protective pathway that acts by the self-ingestion of damaged proteins and organelles (44). Owing to this pathway, minimal levels of energy may be sufficient for cell survival under stress conditions such as I/R injury (44, 45). Therefore, enhanced autophagy may be essential for cardioprotection in I/R injury (45, 46). It has been reported that CoQ_10_ increased several proteins responsible for the activity of autophagy such as Atg5, beclin-1, and LC-3II/LC3-I ratio (31). It also has been involved in the regulation of mitochondrial autophagy (through increasing levels of LC3-II, PINK, and parkin) and attenuation of mitochondrial dysfunction (47). Moreover, CoQ_10_ has been shown to increase the expression of ubiquitin proteins in I/R animal models (32). The ubiquitin-proteasome system is important for a cell to degrade its own dysfunctional contents. The system works as follows: dysfunctional substances are tagged by ubiquitin proteins, in a process called ubiquitination. Then, proteosomes recognize these tags and subsequently remove the dysfunctional constituents of the cell (48–50). Importantly, the coupling of ubiquitin to proteasomal activity requires so-called ubiquitin receptors, which recruit the ubiquitinated protein to the proteasome for degradation (51).

In I/R injury, oxidative stress leads to the formation of dysfunctional oxidized proteins, and it is the proteasome (particularly the 20S proteasome), which is primarily responsible for the removal of such hazardous proteins (52). Accordingly, it is not surprising that recent research has shown that inhibiting the proteasome system exacerbates I/R injury (53). In addition, Hu et al. have recently shown that knocking out ubiquilin 1, a ubiquitin receptor, in I/R mice models led to an accumulation of ubiquitinated proteins, ultimately resulting in larger infarct size compared to mice with increased ubiquilin 1 activity, in whom infarct area was smaller (54).

Furthermore, Tian et al. have demonstrated that pharmacological proteasomal inhibition leads to increased activation of protein kinase C delta (PKCδ) and decreased activation of PKCε (53). The changes in the ratios of these two isozymes, through their effects on mitochondrial functions, lead to increased apoptosis and thus exacerbate I/R injury (53, 55–57). In sum, these findings suggest that CoQ_10_, by increasing the levels of ubiquitin proteins, may enhance proteasomal activity, decrease apoptotic activity, and ultimately conserve myocardial cells after I/R injury.

**Figure 4:**
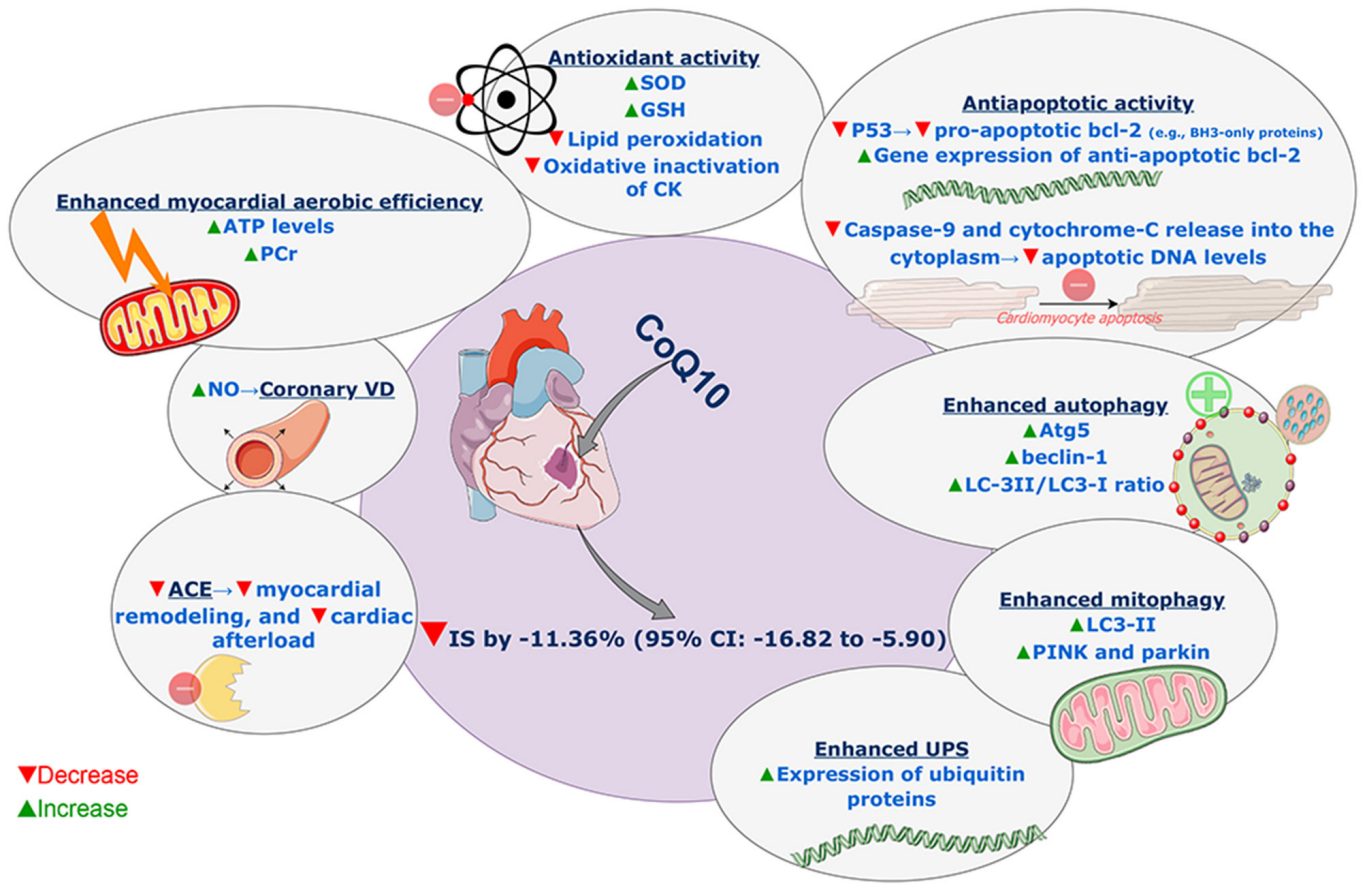
Summary of involved potential mechanisms of action of coenzyme Q_10_ reducing effect on myocardial infarct size in models of myocardial ischemia-reperfusion injury. SOD, superoxide dismutase; GSH, glutathione; CK, creatine kinase; UPS, ubiquitin-proteasome system; ACE, angiotensin-converting enzyme; NO, nitric oxide; VD, vasodilatation; ATP, adenosine triphosphate; PCr, phosphocreatine; CoQ_10_, coenzyme Q_10_; IS, infarct size; CI, confidence interval. This figure was produced on diagrams.net, using SMART materials (Servier Medical Art; smart.servier.com) that are approved under a Creative Commons Attribution 3.0 Unported License.

Coenzyme Q_10_ has also been found to reduce levels of angiotensin-converting enzyme (ACE) in patients following MI (58). This is important for two reasons: first, ACE is a known inducer of remodeling following MI (59); therefore, by reducing ACE levels, CoQ_10_ reduces remodeling and preserves cardiac function. Second, inhibition of ACE can reduce the afterload imposed on the heart, thereby alleviating adverse structural cardiac changes (60). Coenzyme Q_10_ has been found to reduce peripheral resistance and thus afterload (61), which may be partially mediated by its effect on ACE. Supporting this postulate is a meta-analysis showing an attenuated benefit of CoQ_10_ in cohorts using ACE inhibitors (62), which suggests that CoQ_10_ exerts some of its beneficial effects, at least partially, by its effect on the renin-angiotensin-aldosterone system. All involved potential mechanisms of action are summarized in Figure 4.

### Related Evidence From Clinical Studies and Future Prospective

In line with our results, the benefits of CoQ_10_ in terms of cardioprotection/CV prevention in MI (and other CV diseases) have been observed in clinical studies; however, its effect on the infarct size has not been investigated in humans. In a double-blind randomized controlled trial (RCT), Singh et al. (58) assessed the effects of CoQ_10_ (120 mg/day for 24 weeks), compared with placebo, on parameters of left ventricular remodeling in 55 patients with post-MI LVEF <50%. This study revealed that CoQ_10_ significantly reduced the wall thickness opposite the infarction site from (mean [SD]) 12.2 (2) to 10 (1.8) mm compared with placebo (*p* < 0.01). It also significantly suppressed changes in the sphericity index and wall thickening at the infarction site. Huang et al. (63) reported that higher plasma levels of CoQ_10_, measured 1 month after primary PCI, were associated with better left ventricular performance/remodeling after 6 months of follow-up in 55 patients with ST-segment elevation MI (STEMI). Low plasma levels of CoQ_10_ have been observed in patients with cardiomyopathy (17, 64). In a cohort of 236 patients with chronic HF, lower levels of CoQ_10_ were associated with the increased risk of all-cause mortality (hazard ratio [HR]: 1.99; 95% CI: 1.21–3.30, *p* = 0.007) (18). In patients with CV disease admitted to the coronary care unit, low plasma CoQ_10_ (less than 0.59 mg/L, or 0.46 mg/L) was an independent predictor of both in-hospital and long-term mortality (65, 66). In another RCT including 144 patients with AMI, CoQ_10_ (120 mg/day) was compared with placebo for 28 days in terms of CV prevention (67). In comparison with placebo, CoQ_10_ significantly reduced total cardiac events (15 vs. 30.9%, *p* = 0.02), angina pectoris (9.5 vs. 28.1%, *p* < 0.05), total arrhythmias (9.5 vs. 25.3%, *p* < 0.05), and poor left ventricular function (8.2 vs. 22.5%, *p* < 0.05). In 2003, Singh et al. (68) also compared, in a double-blind RCT, the effect of CoQ_10_ (120 mg/day) with vitamin B for 1 year on CV events in 144 patients with recent AMI. Coenzyme Q_10_ significantly reduced the total cardiac events (24.6 vs. 45.0%, *p* < 0.02) and non-fatal MI (13.7 vs. 25.3%, *p* < 0.05) compared with vitamin B. In a meta-analysis of 8 clinical trials with 327 patients undergoing cardiac surgery with cardiopulmonary bypass, CoQ_10_ (30–600 mg/day) for 12 h to 14 days before surgery significantly reduced inotropic drugs requirement and incidence of ventricular arrhythmias after surgery, with no significant effect in terms of cardiac index, the incidence of atrial fibrillation, or duration of hospital stay (69). In a recent Cochrane review that included 11 RCTs with 1,573 patients with HF, CoQ_10_ reduced all-cause mortality (risk ratio [RR]: 0.58; 95% CI: 0.35–0.95) and HF-related hospitalization (RR: 0.62; 95% CI: 0.49–0.78) compared with the control group (70). A significant improvement in LVEF was also observed with CoQ_10_ supplementation in comparison with the control group (MD: 1.77; 95% CI: 0.09–3.44) (70). In a cohort of 443 Swedish healthy elderly individuals, CoQ_10_ (200 mg/day) combined with selenium (200 μg/day) for 4 years resulted in reduced CV death compared with placebo (5.9 vs. 12.6%; *p* = 0.015), a favorable effect that persisted for 10 years after the intervention (HR: 0.51; 95% CI: 0.36–0.74, *p* = 0.0003) (71, 72). Multiple RCTs reported, in line with the previous results, significant improvements in the quality of life of chronic HF patients with CoQ_10_ supplementation (alone or combined with other micronutrients) compared with placebo (73, 74).

Myocardial infarct size has been identified as an important prognostic parameter in MI (10). In a patient-level meta-analysis including 2,632 patients from 10 randomized primary PCI trials, myocardial infarct size measured within 1 month after PCI was significantly associated with the increased risk of all-cause mortality (HR: 1.19; 95% CI: 1.18–1.20, *p* < 0.0001) and hospitalization for HF (HR: 1.20; 95% CI: 1.19–1.21, *p* < 0.0001) for every 5% increase. Therefore, infarct size reduction may be a clinically plausible explanation for the above-mentioned promising results of CoQ_10_ as a cardioprotective agent.

Experimental studies explored the cardioprotective potential of numerous antioxidant agents (e.g., vitamins C and E, N-acetyl cysteine, and allopurinol) based on the central role of oxidative stress in myocardial I/R injury (75, 76). In fact, several preclinical studies have shown promising results with these agents. For example, Ferrari et al. (77) reported that vitamin E infusion in isolated rabbit hearts (20 min before hypoxia) decreased the depletion of ATP and CP and preserved the mitochondrial function and the myocardium ultrastructure. A combination of vitamins C and E reduced infarct size in ischemic, reperfused pigs' heart by LAD artery ligation for 45 min followed by 3 days of reperfusion (78). N-acetyl cysteine (a glutathione precursor, 100 mg/kg), given 2 h after LAD artery ligation followed by 2 h of reperfusion in dogs, significantly reduced ventricular arrhythmias and myocardial infarct size (37 [12.6%]) compared with the control group (55 [7.0%]) (79). Allopurinol, a xanthine oxidase inhibitor, was reported to enhance coronaries relaxation (80) and limit myocardial infarct size in dogs (81, 82) and also in rats (83). Despite these positive results of antioxidants in animal studies, results from large-scale clinical studies on cardioprotection were disappointing (84). Long-term supplementation of vitamins E and C in clinical trials did not show benefit in terms of CV prevention (85–89). In a relatively large RCT that included 251 patients with STEMI undergoing PCI, N-acetyl cysteine did not show any clinical benefit in terms of CV prevention or with respect to myocardial I/R injury limitation compared with placebo (90). As for allopurinol, multiple small trials have shown positive cardioprotective results (e.g., decreased incidence of arrhythmia and improved myocardial efficiency and cardiac function) in patients undergoing coronary artery bypass grafting and in cardiomyopathy (91–93). However, other several trials failed to show benefit (94–97). It has always been a challenge to translate the results of experimental animal studies to human clinical studies (98). Lack of reproducibility of experimental studies, methodological defects (e.g., selection and performance bias), and large disparities (e.g., presence of comorbidities, different cardioprotective endpoints [i.e., infarct size vs. mortality rate] and inconsistency in dosing and timing of the intervention) in study design between animal experiments and clinical studies are possible causes of translational failure (84, 99, 100). Although low methodological quality may apply to the included animal studies, the combination of consistent evidence derived from animal and clinical studies suggests an important role for CoQ_10_ as a cardioprotective molecule following MI. However, large well-designed RCTs with longer durations of follow-up are warranted to further assess the potential cardioprotective benefits of CoQ_10_ in MI.

Animal studies play a critical role in understanding molecular mechanisms in a variety of diseases. However, there are large anatomical and physiological differences between used animal species and humans, especially with smaller animal models (101). Most of the included studies in this meta-analysis are based on small animal models. Thus, more high-quality animal studies on CoQ_10_'s cardioprotective effects in larger models of myocardial I/R injury are still needed for better assessment of the suitable dosing and timing of CoQ_10_ and understanding of the involved mechanisms of action (101). Human equivalent doses of CoQ_10_ (based on body surface area) that ranged from 9.7 to 233 mg/day, for an adult person weighting 60 kg, were used in the included studies (102). However, higher doses of CoQ_10_, preferably given through intravenous or intracoronary routes, should be considered/assessed in future studies for multiple reasons. First, because of its relatively high molecular weight (863.34 g/mol) and insolubility in water, poor oral bioavailability has been a limitation for CoQ_10_ supplementation, which may become more evident in large MI complicated by peripheral hypoperfusion (23, 103). In rats, only a small part of orally supplemented CoQ_10_ was found to reach the circulation, spleen, and liver with none reached the heart or kidney (104, 105). However, CoQ_10_ as a lipid microsphere given intravenously reached both the heart and kidney as well as other tissues in rats (104, 106). Second, CoQ_10_ supplementation was found to be highly safe (107). In patients with Parkinson's disease, doses of 1,200 mg/day, and even 2,400 mg/day were well tolerated, compared with placebo (108). Finally, statins are commonly used drugs in patients with IHD that have been observed to additionally reduce CoQ_10_ levels (109). Therefore, considering higher doses of CoQ_10_ in patients on statins is reasonable. According to the included studies, pretreatment with CoQ_10_ for 3–30 days before induction of I/R injury seems to be favorable for prophylaxis of MI. Other timings, which may be more applicable in patients with unpredictable acute event (i.e., before or at early reperfusion), should be adequately assessed in future preclinical studies (3). Nevertheless, as mentioned before, higher doses of CoQ_10_ administered through intravenous or intracoronary routes may be needed in these timings to effectively increase the heart concentrations of CoQ_10_ (21). More animal studies on CoQ_10_ cardioprotective potential with a background of other comorbidities (e.g., hypertension, obesity, and diabetes mellitus) are needed for a better clinical insight. In addition, proof-of-concept clinical trials should include infarct size as an endpoint when assessing CoQ_10_'s cardioprotective effects (10).

### Limitations

Our meta-analysis has some limitations. First, significant heterogeneity was observed in the current analysis. Variability in animals' characteristics and methodological differences (e.g., model type, risk of bias sources, and method of ischemia) among the included studies may explain this heterogeneity. Nevertheless, random-effects model and subgroup analyses were applied to address this heterogeneity. Second, potential publication bias was suggested by funnel plot visual inspection. However, the *trim and fill* approach was used to adjust for this bias. Third, most of the included studies were based on smaller animal models, which are less similar to humans compared with larger ones. Fourth, data on cardiac function were not adequately reported in the included studies. Fifth, all included studies did not report any information about multiple domains of bias assessment (e.g., sequence generation and allocation concealment). Sixth, infarct size assessments were short-term, ranging from 45 min to 72 h after reperfusion. Finally, all included animals were without CV comorbidity, not reflecting cases of MI in clinical practice that may have multiple CV risk factors (e.g., obesity, diabetes mellitus, and hypertension).

## Conclusion

Coenzyme Q_10_ significantly decreased myocardial infarct size by 11.36% compared with the control group in animal models of myocardial I/R injury. Additionally, this beneficial action was retained regardless of model type (either *in vivo* or *ex vivo*) and reperfusion time (either ≤ 4 h or >4 h). Significant improvements in cardiac function parameters were also observed with CoQ_10_. High-quality large animal studies are still needed to confirm these results and to further explore the involved mechanisms. Moreover, these results provide the rationale for future large well-designed RCTs with longer durations of follow-up to assess their translation into clinical application.

## Data Availability Statement

The original contributions presented in the study are included in the article/Supplementary Material, further inquiries can be directed to the corresponding author/s.

## Author Contributions

KA helped in literature search, screening, data extraction, data analysis, and manuscript writing. AS helped in screening, data extraction, and manuscript writing. MB helped in study design, supervision, coordination, manuscript writing, and revision. All authors have approved the final article.

## Conflict of Interest

The authors declare that the research was conducted in the absence of any commercial or financial relationships that could be construed as a potential conflict of interest.

## Publisher's Note

All claims expressed in this article are solely those of the authors and do not necessarily represent those of their affiliated organizations, or those of the publisher, the editors and the reviewers. Any product that may be evaluated in this article, or claim that may be made by its manufacturer, is not guaranteed or endorsed by the publisher.
